# Is Multiple Sclerosis an Autoimmune Disease?

**DOI:** 10.1155/2012/969657

**Published:** 2012-05-16

**Authors:** Bharath Wootla, Makoto Eriguchi, Moses Rodriguez

**Affiliations:** ^1^Departments of Neurology, Mayo Clinic, 200 First Street SW, Rochester, MN 55905, USA; ^2^Division of Neurology, Saga University Faculty of Medicine, Saga 849-8501, Japan; ^3^Department of Internal Medicine and Advanced Comprehensive Functional Recovery Center, Saga University Faculty of Medicine, Saga 849-8501, Japan; ^4^Departments of Immunology, Mayo Clinic, 200 First Street SW, Rochester, MN 55905, USA

## Abstract

Multiple sclerosis (MS) is an inflammatory demyelinating disease of the central nervous system (CNS) with varied clinical presentations and heterogeneous histopathological features. The underlying immunological abnormalities in MS lead to various neurological and autoimmune manifestations. There is strong evidence that MS is, at least in part, an immune-mediated disease. There is less evidence that MS is a classical autoimmune disease, even though many authors state this in the description of the disease. We show the evidence that both supports and refutes the autoimmune hypothesis. In addition, we present an alternate hypothesis based on virus infection to explain the pathogenesis of MS.

## 1. Introduction

Studies using imaging, serology, pathology and genetics, and patient response to anti-inflammatory treatments indicate that multiple sclerosis (MS) is primarily an inflammatory demyelinating disease of the central nervous system (CNS) with varied clinical presentations and heterogeneous histopathological features. The disease has a peak onset between ages 20 and 40 years [1]; however it may also develop in children and in addition has been reported in individuals aged above 60 years. MS affects women approximately twice as often as men [2–5]. MS results in a plethora of neurological manifestations and is a leading cause of nontraumatic disability among young adults and has great socioeconomic impact in developed countries [6]. Based on the epidemiological studies, approximately 400,000 people have MS in the United States, with 200 new cases added every week. The pathogenesis of MS remains elusive and there were no definitive cause and no effective cure. Therefore, MS can be classified as an episodic demyelinating disease of the central nervous system. Disease pathophysiology is complex and involves genetic susceptibility, environmental factors, and development of a pathologic immune-mediated response leading to focal myelin destruction, axonal loss, and focal inflammatory infiltrates.

The pathophysiology of MS is further fraught with confusion as researchers struggle to classify the disease as either pathological [7] or clinical [8]. Investigators and clinicians who have studied MS agree that the immune system plays a critical role in the development of lesions, especially during the acute early phases of the disease characterized by relapses. Relapses are fundamentally a manifestation of an inflammatory response occurring mostly in the white matter of the nervous system but also within myelin tracts in the gray matter. This results in focal demyelination with relative axonal sparing. The best evidence for inflammation-induced relapses comes from work in MRI, which demonstrates the association of relapses with gadolinium enhancement that is disruption of the blood brain barrier. The main pathologic hallmark of MS is the demyelinated plaque, which has specific histological and immunocytological characteristics depending on the activity of the disease [9–12]. Histologically, an MS plaque is characterized by marked predominance of CD8^+^ T cells and a relative lack of CD4^+^ T cells (ratios of 100 : 1 to 50 : 1). In addition, there is a sea of macrophages, which may have a primary role in engulfing myelin debris. Whether they are also primary effectors in the disease process is unknown. Another important immunopathological feature is continuous synthesis of immunoglobulins (oligoclonal IgG's) in cerebrospinal fluid (CSF). The evidence associating antibodies with MS derives from studies such as by Kabat et al., who described increased levels of immunoglobulin (Ig) in the cerebrospinal fluid (CSF) [13]. CSF IgG and oligoclonal bands remain the most predictive immunological test for the diagnosis of MS. All immunoglobulin subtypes have been implicated in MS. The underlying immunological abnormalities lead to presentation of different autoimmune manifestations.

## 2. Is MS an Autoimmune Disease?

From most references gleaned in the literature, MS is boldly stated as an autoimmune disorder. However, the evidence for such a statement is weak and circumstantial. We have updated and revised criteria for determining whether a disease is autoimmune in nature [14]. The main criterion of a given autoimmune disease is that a precise autoantigen be present in all patients with the disease. Despite multiple attempts to identify various proteins, lipids, and gangliosides in myelin as potential MS antigens, none have been proven or confirmed. Secondly, administration of autoantibody or T cells induces autoimmune disease in normal animals. These approaches have been attempted in animal models of MS with contrasting results [15, 16]. A third criterion is the ability to induce lesions by immunizing animals with relevant autoantigen. This had been partially achieved but with problems. The fact that multiple different antigens can induce the disease process in animal models without one specific antigen being superior to the other makes the results ambiguous from the standpoint of identifying the relevant antigen. The fourth criterion is the ability to isolate autoantibody or autoreactive T cells from the lesion or from serum. Many investigators have suggested a higher precursor frequency of T cells, specifically of the CD4 subgroup, in patients with MS when compared to healthy controls, which recognize MBP, proteolipid protein (PLP), MOG, or other such antigens from myelin. Unfortunately, because similar positive results are obtained from normal individuals, this criterion is not satisfied. The fifth criterion is the correlation between the autoantigen or the autoreactive T cells with disease activity. Autoreactive T cells occur with greater frequency in patients experiencing an exacerbation than in patients with progressive disease, which suggests a possible correlation between auto-reactive T cells and disease activity. Even though the precursor frequency of autoreactive T cells may be higher in MS than in normal controls, the presence of autoreactive T cells demonstrated in normal controls makes a definitive conclusion about MS as autoimmune more difficult to accept. The sixth criterion is the presence of other autoimmune disorders or autoantigens associated with the disease. This issue has been addressed by a number of investigators, and there have been occasional case reports demonstrating the presence of MS with other autoimmune diseases, for example, myasthenia gravis [17] and diabetes mellitus [18]. However, population-based cohort studies performed in the Olmsted county, Minnesota, failed to show any association between autoimmune diseases and MS [19]. The only possible increased odds ratio was found with thyroid disease, when both hyperthyroidism and hypothyroidism were combined. In addition, rare cases have been described in patients with both MS and inflammatory bowel disease [17, 18]. There have also been multiple studies looking at the presence of autoantibodies, a characteristic of patients with autoimmune diseases, such as the antibodies seen in Sjögren's syndrome, systemic lupus erythematosus (SLE), or myasthenia gravis, but to date, no evidence indicates that the presence of these antibodies is greater in MS patients than in normal controls. Of interest, this differs greatly from neuromyelitis optica (NMO) [20, 21], where there is clearly an association between the presence of autoantibodies and NMO (discussed later in this paper).

## 3. Immune Manifestations of MS: Role of Antibodies

There is evidence to suggest that part of the immunopathogenesis of MS is mediated by antibodies. Studies have indicated intrathecal production of antibodies, which occur after clonal expansion manifested by the identification of oligoclonal bands after CSF electrophoresis [22]. Studies at the Mayo Clinic, Austria, and Germany reported on the heterogeneity of MS lesions in CNS tissue and their implications for the pathogenesis of demyelination. A detailed immunohistochemical study was performed in active MS lesions from 83 biopsies and autopsies of MS patients, following, which were identified four different pathologic subtypes of active MS lesions. One of the subtypes, Pattern II, demonstrated the presence of macrophages and T cells but, in addition, a prominent display of antibodies and complement [9]. This data provided evidence that lesion patterns were heterogeneous among patient subgroups but homogeneous in the same patient. Barnett and Prineas studied acute MS lesions and found complement activation, oligodendrocyte apoptosis, and remyelination, findings that overlapped the Mayo/Germany/Austria studies [23]. A recent study identified activated complement (C3d and C9neo) on fragmenting myelin sheaths in the outer actively demyelinating lesions in 20 patients with relapsing MS (58/58 active lesions) [24]. The authors reported the presence of activated complement on disintegrating sheaths in diverse diseases affecting white matter, including viral and autoimmune encephalitis, NMO, and even ischemic infarcts, suggesting that this phenomenon is not limited to MS. In a more recent study, Breij et al. [25] investigated to what extent the four pathological pattern criteria translated to active lesions from patients with established MS. These authors concluded that MS lesions displayed a homogenous profile. The authors were unable to confirm the lesion heterogeneity or interindividual heterogeneity with respect to Ig and complement immuno-reactivity. However, it is possible that all the lesions studied were not active. It is possible that the heterogeneous features reported in active MS lesions that were sampled at varied time-points are the evolution of a single pathophysiological process, rather than discrete immunopathogenic patterns. This may be the case, or the majority of active lesions will present with Pattern II phenotype, as supported by findings of a study by Barnett and Sutton, where the authors described that in 22 patients drawn from a large unselected pool of MS material, 33 actively demyelinating lesions presented Pattern II pathology [26]. The four-pattern system has not been completely independently verified to date because of the lack of available highly comparable pathologic material.

The response to plasma exchange (PLEX) in acute fulminant MS provides further evidence for the role of immunoglobulin or serum components in the disease. Rodriguez et al. demonstrated conclusively for the first time the detrimental effect of plasma components in inflammatory demyelinating diseases of the CNS; PLEX in acute episodes of fulminant CNS inflammatory demyelination, which did not respond to high-dose methylprednisolone, led to a marked neurologic improvement in 6 patients [27]. These results were confirmed in a randomized, sham-controlled, double-masked study of PLEX without concomitant immunosuppressive treatment in patients with recently acquired, severe neurological deficits resulting from attacks of inflammatory demyelinating disease, who failed to recover after treatment with intravenous corticosteroids [28]. A retrospective study investigated 19 patients treated with PLEX for an attack of fulminant CNS inflammatory demyelinating disease. All patients with pattern II (*n* = 10), but none with pattern I (*n* = 3) or pattern III (*n* = 6), achieved moderate to substantial functional neurological improvement after PLEX (*P* < 0.0001) [29]. The fact that all cases, which responded to PLEX, had a biopsy demonstrating Igs and complement, whereas none that responded showed this immunologic pattern, provided the strongest proof that the pathologic patterns are unique and have therapeutic significance. Numerous publications during the last few decades have supported the idea that CSF oligoclonal bands correlate to the level of B-cell involvement in MS [30]. In addition, evidence indicates that oligoclonal bands may have a prognostic value. One prospective study of patients with acute isolated demyelinating episode demonstrated intrathecal immunoglobulin synthesis to be a better predictor of MS progression than MRI [31]. Another prospective study showed that presence of CSF oligoclonal bands in early MS generally correlated with a worse outcome [32]. A recent study showed strong correlation between levels of oligoclonal bands (OCBs) and prognosis for MS disability [33].

## 4. Antigen: Specificity of Antibodies Found in MS

After several years of research, confirmation of the antigen-specificity of antibodies in MS is still lacking. Due to their broad reactivity, IgG in CSF of patients with MS may represent synthesis of “nonsense” antibodies irrelevant to pathogenesis [34–36]. However, other experiments found molecular uniformity and temporal persistence of the Ig response in MS, thus conflicting with the nonsense antibody proposal [37]. It is possible that relevant antigens are limited to the myelin sheath. Studies demonstrated the serological and/or CSF presence of antibodies directed against MBP and/or myelin/oligodendrocyte glycoprotein (MOG) in patients with MS [38]. However, myelin-specific antibodies are not limited to MS. Using an enzyme-linked immunosorbent assay, Karni et al. compared levels and frequencies of anti-MOG antibody between patients with MS, patients with other neurological disorders, and healthy control subjects [39] and found minor differences. In a parallel line of research, some reports suggested lipids or carbohydrates as possible candidate antigens for the humoral immune response [40, 41]. Anti-alpha-glucose-based glycan IgM antibodies have been suggested to be predictors of relapse activity in MS after the first neurological event [42]. Others suggested that serum anti-Glc(alpha1, 4)Glc(alpha) antibodies serve as biomarkers for relapsing-remitting MS [43]. Antibodies to myelin proteins, lipids, and carbohydrates can be extracted from the tissue and sera of some MS patients.

## 5. Immune Manifestations of MS: Role of T Cells

### 5.1. CD4^+^ T Cells as Initiators of Disease versus Effectors in Destruction of Myelin

**Figure 1:**
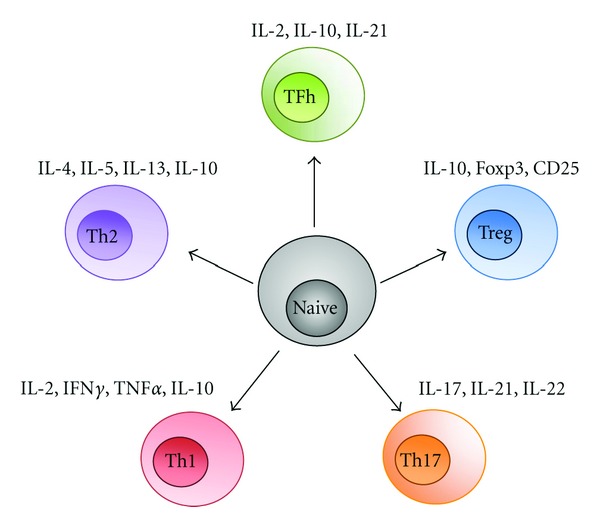
CD4^+^ T Cells differentiated into subsets. CD4^+^ T cells can differentiate into different subtypes based on the factors within the inflammatory milieu with which T cells come into contact. TH1^+^ cells secrete IFN-*γ* and tumor necrosis factor (TNF) and mediate the pathology in experimental autoimmune encephalomyelitis (EAE). Many of the results previously attributed to TH1 cells are actually mediated by Th17^+^ cells. Th17^+^ cells secrete IL17, IL21, and IL22. These cells have been identified in MS lesions, where they may serve as important effectors. As a result of  TGF-*β* stimulation, CD4^+^ T cells develop into T-regulatory cells. These cells downregulate the immune response and express FOXP3, CD25, and IL10. The mechanism of suppression is by the secretion of factors such as IL10. TH-helper cells provide help to other T cells, such as CD8^+^ T cells or B cells. Th-helper cells secrete IL2, IL10, and IL21. Th2^+^ cells downregulate the immune response and are associated with recovery from acute attacks in EAE and, possibly, MS. The cytokines that mediate the downregulation of the immune response are IL4 and IL10, in addition to IL13 and IL5.

The area of greatest confusion in the MS literature concerns the role of CD4^+^ T cells in disease pathogenesis. CD4^+^ cells predominate in experimental autoimmune/allergic encephalomyelitis (EAE) as the effectors that induce disease and destroy myelin. Therefore, due to the influence of the experimental model, many investigators have attempted to show that CD4^+^ T cells also play a pathogenic role during the evolution of MS. Unfortunately, many findings regarding the role of CD4^+^ T cells reported have not been reproduced elsewhere [44]. However, there is strong experimental evidence that any immune response must begin through the engagement of the antigen recognized by receptors on CD4^+^ T cells. In concept, dendritic cells, both outside and/or inside the CNS, take up the exogenous or endogenous antigen and present it to CD4^+^ T cells. As a result, these CD4^+^ T cells differentiate into four distinct subtypes depending on the inflammatory milieu (Figure 1). The first is the Th1-type CD4^+^ T cell, which primarily secretes IFN-*γ* and TNF-*α*. The second is a CD4^+^ T cell frequently called Th2, which secretes primarily TGF-*β* and IL-10. The third is a CD4^+^ TREG cell that performs a regulatory function [45]. These T cells express a number of transcription factors including FoxP3 and other molecules [46, 47]. These cells play a major role in downregulating the immune response [48–50]. Finally, there are CD4^+^ T cells that primarily secrete IL17 called Th17 cells. These Th17 cells induce most of the pathology in EAE. There is evidence of their presence in the MS plaque, where they may preferentially recruit IFN-*γ* [51]. Data suggest that CD4^+^ T cells from MS patients use unique human T-cell beta-receptors [52]. In these studies, the investigators used T-cell lines from MS patients as well as healthy controls and showed that these CD4^+^ T-cell lines reacted against specific human myelin basic proteins, the first being residues 84–102 and the second being residues 143–168. They showed that the CD4^+^ T-cell receptors being used were primarily of the V*β*17 and V*β*12 family. V*β*12 receptors were used frequently in recognition with the MBP (84–102) peptide, while V*β*17 mostly reacted against MBP (143–168).

The presence of unique T-cell receptor V-*β* gene usage has generated a series of experimental animal trials as well as early human trials with the goal of deleting specific V-*β* T cells in MS patients [53]. These experiments have been relatively successful in EAE; however, the approach has been less effective in human patients. Of interest, investigators have also isolated MBP-reactive CD4^+^ T-cell lines from normal human blood [54]. The fact that these T-cell lines respond to MBP [55], similarly to what is observed in MS patients, has raised major questions as to the specificity of the response of CD4^+^ T-cell lines to myelin antigen in MS patients [56]. These CD4^+^ T-cell lines obtained from non-MS patients secreted IL2 similar to that seen with MS patients. All of the T-cell lines isolated from the peripheral blood were of the CD4 phenotype [57]. Investigators have examined peripheral blood lymphocytes from MS patients and other neurological controls in effort to study specific T-cell populations against purified human MBP and other brain antigens [58]. Investigators showed that lymphocytes from MS patients were more likely to react against MBP. Unfortunately, they discerned only minor differences between MS patients and normal controls as to the specificity of the response to any brain tissue antigens. The majority of responses were found in patients with chronic progressive MS, a phase when T cells are least active in the disease. These results also have raised concerns about the specificity of the T-cell immune response to myelin antigens in MS patients given the not easily discernible differences between MS patients and normal individuals. Even those investigators claiming to show a positive, “statistically significant” response show such an overlap in the results between patients and controls that these assays have never been developed as a diagnostic test for MS [59].

Recent work has focused on Th17^+^ cells in MS [60, 61]. Investigators looked at evidence implicating IFN-*γ* producing hybrid T cells (so-called Th1 cells as well as IL17^+^ lymphocytes (Th17^+^ cells)) in MS. They compared this to animals with EAE and demonstrated expansion of Th17 lymphocytes from the blood of healthy controls as well as from patients with relapsing MS. In response to IL23, which is known to expand the Th17 phenotype, they showed simultaneous expressions of IFN-*γ* and IL17. They noted that patients with relapsing-remitting MS had increased production of IFN-*γ* by Th17 cells. The same findings were also present in the experimental model. Both these data sets support the hypothesis that Th17 cells play a role in the pathology of MS and EAE. However, the presence of Th17 cells does not automatically prove that they play a role in pathogenesis [62]. No data is available that deletion of Th17 cells improves MS or that elevated Th17 cells in lesions correlate with disability. This is in contrast to the work done on CD8^+^ T cells (discussed hereinafter), which reveals a strong correlation between CD8^+^ T cells, perforin, and other molecules as secreted by CD8^+^ T cells with disease disability.

### 5.2. CD8^+^ T Cells: Primary Mediator of Effector Function in the MS Plaque

Pathological studies demonstrate that the CD8^+^ T cell is the most common T cell observed in the MS plaque. Conventional perception is that CD8^+^ T cells have two major functions: cytotoxicity and suppression. In MS, because of the strong bias of the experimental autoimmune encephalomyelitis (EAE) models, the CD8^+^ T cell has been primarily thought to play a suppressive role. In EAE, the CD4^+^ T cell, through its Th1 and Th17 function, mediates the disease and induces the inflammatory response, neurological deficits, paralysis, and histological findings. In EAE, CD8^+^ T cells are associated with recovery of neurologic function and have been shown to have suppressive properties. In contrast, in the MS plaque, the CD8^+^ T cells appear to play a much more aggressive role rather than just suppressing the inflammatory response. CD8^+^ T cells interact with major histocompatibility (MHC) class I antigens to induce their response. In normal CNS, class I MHC is observed only in vascular cells and rare meningeal cells. However, in the midst of an inflammatory process such as MS, class I MHC is observed in astrocytes, oligodendrocytes, and neurons and even rarely on axons [63]. In addition, the CD8^+^ T cells correlate with axonal injury, and there is strong evidence *in vitro* that CD8^+^ T cells play a major role in transecting axons [64, 65]. Relapses of MS are associated with increased CD8^+^ T cell cytotoxicity in the CSF [66]. A number of clinical trials with monoclonal antibodies, specifically against CD4^+^ T cells [67], failed to show any therapeutic benefit in MS as opposed to broader spectrum antibodies (alemtuzumab CD52), which are able to deplete all T cells [68], including CD8^+^ T cells. It is also important to emphasize that CD8^+^ T cells may play a major role in a number of proven autoimmune disease including SLE, diabetes mellitus, Crohn's disease, Graves' disease, and autoimmune Addison's disease [63].

**Figure 2:**
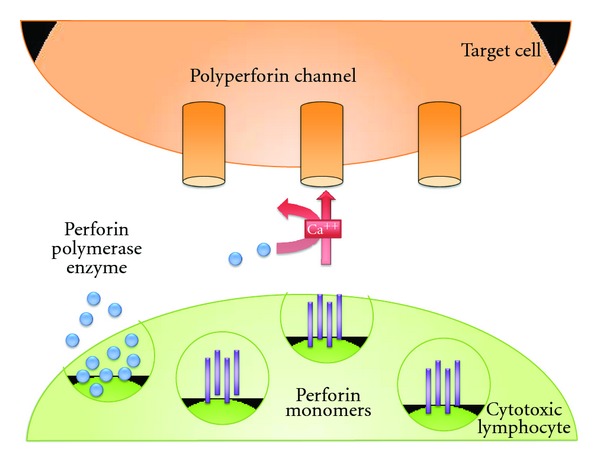
Perforin is the primary mediator of  injury by CD8^+^ T cells. Perforin is the primary molecule known to mediate injury by CD8^+^ T cells. Perforin mediates axonal transection in multiple sclerosis (MS) and correlates with neurological disability. Cytotoxic T cells secrete perforin in the form of granules along with granzymes. This release activates calcium, which results in “poly-perforin” channels on the target cells. This results in holes in the membrane of the target cells, causing leakage of intracellular material, which results in cell death.

Finally, CD8^+^ T cells show oligoclonal expansion in MS brains, blood, and CSF that have not been reported with CD4^+^ T cells [69–71]. Some of the cytotoxic T cells react against autoantigens such as myelin-basic protein [72]. If these cells traveled randomly in the CNS, then presumably their CD3 junction region length would show a normal Gaussian distribution. In contrast, there is skewing of the CDR3 junction regions in MS, suggesting a selective infiltration or expansion of CD8^+^ T cell clones into the CNS [71]. Moreover, the T-cell receptors of the CD8^+^ T cells demonstrate distinct CD8^+^ T-cell clones with conserved specificity implying recognition of a similar antigen that results in their proliferation in the CNS. Much effort has focused on the potential role of IL17 in the MS plaque, and because of the bias of experiments in EAE, it has been proposed that this comes from the CD4^+^ T cells. However, there is evidence that IL17 is also made by CD8^+^ T cells [73]. Defining IL17^+^ CD8^+^ T cells opens up new avenues for future research and new targets from the standpoint of immunotherapy. CD8^+^ T cells secrete a number of molecules including granzymes and perforin. Strong evidence suggests that perforin contributes to axonal injury in the MS plaque (Figure 2). The presence of perforin correlates with neurologic disability and with the presence of “black holes” on MRI. Therefore, CD8^+^ T cells play a critical role during the acute inflammatory phase of the disease as well as during the neurodegenerative phase. CD8^+^ T cells account for axonal damage in MS as well as long-term neurological deficits. There is evidence that CD8^+^ T cells play a major role in the secondary progressive phase of the disease by the secretion of lymphotoxin [74]. In studies of cytokine secretion in patients with secondary progressive MS and normal controls, investigators found clear evidence of anti-CD3-stimulated CD8^+^ T cells in the patients with secondary progressive MS. These cells secreted lymphotoxin and other cytokines, which play a critical role in the evolution of the progressive phase of the disease. This provides strong evidence that the CD8^+^ T cell plays a role in the neurodegenerative aspect of the progressive phase of the disease as well as in the early acute phase.

It is also important to emphasize a possible regulatory role for CD8^+^ T cells in MS. Investigators have identified CD8^+^/CD25^+^ Foxp3^+^ as regulatory T cells in MS patients [75]. In these studies, they examined the peripheral blood, CSF, and CD8^+^ T cell clones from patients with MS exacerbations, patients with remissions, healthy individuals, and patients with other inflammatory neurological diseases. The inhibition of CD4^+^ self-reactive T-cell proliferation by CD8^+^ regulatory cells was mediated by IL10 and transforming growth factor beta (TGF-*β*). Any attempt to delete CD8^+^ T cells from the MS lesion could potentially worsen the disease by eliminating regulatory cells. Therefore, caution must be taken in any effort to manipulate the CD8^+^ population.

## 6. Autoimmunity-Based Evidence for NMO Pathogenesis

Hinson et al. [76–78] discovered the occurrence of anti-AQP4 IgG in patients with NMO. It was further demonstrated that AQP4-reactive antibodies appear in the pathologic lesions [79] and that levels of AQP4 antibody and disease activity were correlated [80]. NMO is associated with other autoimmune disorders [20]. In addition, autoantibodies against other common autoimmune diseases, such as Sjogren's syndrome and systemic lupus erythematosus, appear in the serum of NMO patients [21] but not in the serum of MS patients [19]. Given the autoimmune hypothesis associated with NMO, we hypothesized that PLEX, a conventional method to remove circulating autoantibodies in patients, would be beneficial. Interestingly, PLEX proved to be a highly successful treatment for NMO arguing in favor of an immune-mediated pathogenesis of this disease [81]. In line with the autoimmune-mediated hypothesis, humoral immunity-suppressing drugs such as mitoxantrone hydrochloride [82] (a synthetic anthracenedione that was approved for the treatment of worsening relapsing-remitting and secondary progressive MS), mycophenolate mofetil [83] (an immunosuppressive therapy), and rituximab [84] (a B-cell depleting therapy) were demonstrated to be beneficial for treatment of NMO. De Parratt and Prineas recently described an abrupt destruction of perivascular astrocytes in patients with NMO that preceded oligodendrocyte apoptosis in early lesions. Their findings add to the experimental evidence that serum antibody directed against astrocytes present in a high proportion of patients with NMO is pathogenic. In addition, their data supports a new definition of the disease based on pathology: *NMO is a demyelinating disease characterized pathologically by multifocal lesions disseminated in time and space and in which demyelination is secondary to acute destruction of perivascular astrocytes* [85]. However, Takano et al. reported that astrocytic damage is far more severe than demyelination in NMO [86]. It is now considered that NMO is an inflammatory autoimmune disorder of the CNS.

## 7. An Alternate Hypothesis for MS Pathogenesis

An attractive hypothesis to explain the immune-mediated pathogenesis of MS is that it is induced by an infectious agent. Even though no infectious agent has convincingly been demonstrated in MS, there is experimental evidence to support the hypothesis.

## 8. Experimental Evidence for Virus-Induced Demyelination

Experimental infection of laboratory animals with various viruses induces demyelination in the CNS. The most studied viral animal model of MS is the disease induced by Theiler's murine encephalomyelitis virus (TMEV), a mouse enteric pathogen that belongs to the single-stranded RNA picornaviruses [87]. The disease model is chronic-progressive in susceptible mice, a striking contrast to the much-used autoimmune EAE model. Two salient features make it the best-suited model for studying MS. There is evidence of an immune response to virally infected cells [88, 89] as well as autoimmune response triggered by viral infection in the CNS [90], both of which are potentially similar to MS. Miller et al. reported that TMEV infection leads to CNS autoimmunity via epitope spreading [91]. TMEV infection of oligodendrocytes results in cell lysis and liberation of more virions [92]. On the contrary, infection of TMEV in macrophages is restricted and results in their apoptosis. Virus spreads from macrophages to other macrophages and oligodendrocytes, adding to the immunopathological destruction of myelin. Demyelination is in part the result of direct virus destruction of oligodendrocytes but also the consequence of immune and inflammatory responses. Other viral models of demyelination include mice with JHM and MHV-4 virus (coranoviruses) infection, dogs with canine distemper virus, and sheep and goats with Visna virus and caprine arthritis-encephalitis virus. An animal model of virus-induced demyelination with no relapses is the Semliki Forest virus (SFV) infection of mice [93]. All viruses are capable of establishing persistent viral infection over a long period without inducing mortality of the host. All of these examples make a case for the viral hypothesis of CNS demyelination.

## 9. Evidence for a Virus-Induced Etiology of MS

Epstein-Barr virus (EBV), human herpes virus 6 (HHV-6), varicella zoster virus (VZV), and Chlamydia pneumonia are some of the proposed infectious agents in humans implicated in MS. Many studies have demonstrated antibody titers to a broad range of pathogens in MS patients; however, many of these findings remain solitary and unconfirmed. EBV is a B-lymphotropic human DNA herpes virus that infects most individuals asymptomatically but causes infectious mononucleosis (IM) in some [95, 96]. Cepok et al. identified EBV proteins as putative targets of the immune response in MS [97]. Another study demonstrated the increased risk of MS in individuals with a clinical history of IM [98, 99]. Recently, researchers from the United Kingdom studied the prevalence of MS and infectious mononucleosis (IM) and how they relate to ultraviolet B (UVB) exposure [100]. As previously shown in other studies, MS highly correlated with IM [101, 102]. As a control, the authors also examined correlations of MS with cytomegalovirus prevalence and varicella prevalence, respectively, both of which were not correlated with MS. Of note, the authors found that UVB in any season correlated closely with MS and EBV infection. These results fit well with the EBV hypothesis because there may be a mechanism through which UVB radiation mediates MS risk.

**Figure 3:**
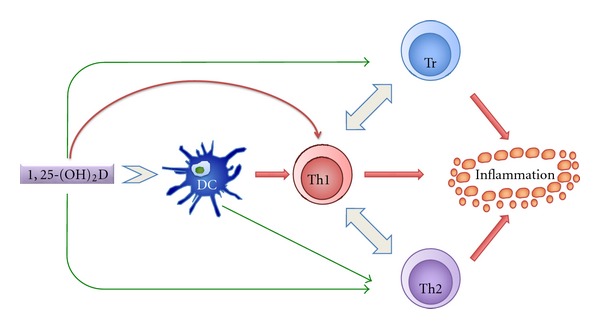
The *in vitro* effects of 1,25(OH)_2_D on the immune system. The effects of 1,25(OH)_2_D either directly or indirectly are depicted by arrows. While a green arrow represents positive influence, a red arrow represents the negative influence. The negative influence on inflammation indicates dampening of the inflammatory response. DC: dendritic cell; Th1: T helper type 1 lymphocyte; Th2: T helper type 2 lymphocyte; Tr: regulatory T lymphocyte [94].

It has been suggested that low vitamin D levels result in immunosuppression that lead to an increase in EBV infection. It is also known that a low amount of UVB decreases vitamin D levels. The geographical variation in the MS prevalence, with a higher prevalence of the disease in northern latitudes and a lower prevalence at the equator, is well established [103–105]. This variation in MS prevalence correlates positively with changes in the serum concentrations of 25-hydroxyvitamin D [106–108]. Several, but not all, studies show an inverse correlation between serum 25-hydroxyvitamin D concentrations and the incidence of MS, the severity, and progression of disease [109–120]. Vitamin D, and its biologically active metabolite 1,25-dihydroxyvitamin D_3 _(1,25(OH)_2_D_3_), not only plays an important role in the regulation of calcium and phosphorus homeostasis but also is an important modulator of immune function. 1,25(OH)_2_D_3_ functions by associating with the vitamin D receptor that is widely distributed in a number of calcium-transporting tissues, neural tissues, and immune cells (dendritic cells, T-lymphocytes, B lymphocytes and macrophages) [121–128]. 1,25(OH)_2_D_3_ increases macrophage activity, inhibits dendritic cell maturation, inhibits B-cell functions, and favors the production of T-helper 2 cells, thereby shifting the ratio of Th1/Th2 cells in favor of Th2 helper cells (Figure 3) [94, 129–134]. The polarization of activated CD4^+^ T cells to a Th-1 phenotype (IL-2, IFN*γ*, TNF*α* secretion) or to a Th-2 phenotype (IL-4, 5, 13, 10 secretion) represents a major determinant of the nature of subsequent cellular and humoral immune responses. It is a self-perpetuating process in that one subtype inhibits the generation of the other [131, 132]. The primary generation of Th-1-type T-cell responses is potently inhibited by 1,25(OH)_2_D_3_ both *in vitro* and *in vivo*. 1,25(OH)_2_D_3_ also induces production of human cathelicidin, LL-37, which is particularly effective against respiratory viruses such as influenza [135]. The lack of vitamin D may result in persistent infection, for example, EBV infection causing IM, thereby leading to a higher risk for MS [136].

## 10. Conclusions

In MS, for an oligodendrocyte to be injured by inflammatory cells, it must express MHC class I or class II genes. CD8^+^ T cells can then engage a novel protein that is expressed in the context of class I MHC. CD8^+^ T cells would then secrete perforin, granzyme, or other factors that may directly injure or kill the oligodendrocyte resulting in demyelination. Antibodies, through molecular mimicry, may recognize autoantigens of the CNS and can also injure the oligodendrocyte by binding to the surface of the cell and, in association with complement, may induce direct injury to myelin or the oligodendrocytes. This partially leads us to the autoimmune hypothesis. In addition, the B cells may also present virus antigens in the context of class II MHC molecules. The oligodendrocyte or microglia itself may also express class II MHC. This presentation of the viral antigen must be processed, which allows the CD4^+^ T cells to be engaged with class II MHC to induce injury, the common mechanism of injury presumed to be present in experimental autoimmune encephalomyelitis. The oligodendrocyte may die as a consequence of direct and persistent virus infection. These mechanisms of injury may be independent or occur concurrently in each brain. All of these mechanisms lead to demyelination that the host may correct by transient remyelination. Ultimately, the demyelination process overtakes remyelination resulting in axonal damage, thus leading to permanent neurologic deficits. At the present time, there is no clear evidence that these patterns of injury relate to various stages of the disease course and do not correlate with the clinical subtypes of relapsing-remitting multiple sclerosis, secondary progressive multiple sclerosis, or primary progressive multiple sclerosis, although this is yet to be determined.

Many of the observed findings have subsequently led investigators to false conclusions regarding MS pathogenesis. Immune cells are present in the MS plaque, and the immune system is important in the pathogenesis of the disease because a number of immunomodulatory and immunosuppressive therapies do decrease relapses and the number of gadolinium-enhancing lesions in MS brain. However, the long-term consequences of immunosuppression on disease course are unknown because most published clinical trials end after two years of observation, an insufficient period of time to address the long-term consequences of these treatments. It is increasingly evident that CD8^+^ T cells and their effector molecules may directly affect the disease process. Unfortunately, despite years of documentation of involvement of CD8^+^ T cells in MS lesions, scant experimentation has been performed on this aspect of the immune response. This is probably due to the bias of the experimental model, EAE, in which CD8^+^ T cells play only a regulatory role whereas CD4^+^ T cells play a major effector role in disease pathogenesis. Once we move away from the experimental model and begin to investigate MS in humans, it becomes apparent that the MHC class II CD4^+^ T-cell immune response yields less important critical data of the MHC class I CD8^+^ T-cell immune response. The most important diagnostic test for MS continues to be the presence of increased CSF IgG and the presence of specific oligoclonal bands in the CSF but not in the serum. Therefore, it is critical to identify the specificity of these bands. Ultimately, it may be proven that CSF oligoclonal IgG bands play a neuroprotective rather than a pathologic role [137–140].
